# Lightweight Depth Completion Network with Local Similarity-Preserving Knowledge Distillation

**DOI:** 10.3390/s22197388

**Published:** 2022-09-28

**Authors:** Yongseop Jeong, Jinsun Park, Donghyeon Cho, Yoonjin Hwang, Seibum B. Choi, In So Kweon

**Affiliations:** 1 The Robotics Program, Korea Advanced Institute of Science and Technology, 291 Daehak-ro, Yuseong-gu, Daejeon 34141, Korea; 2 School of Computer Science and Engineering, Pusan National University, 2 Busandaehak-ro 63beon-gil, Geumjeong-gu, Busan 46241, Korea; 3 Department of Electronics Engineering, Chungnam National University, 99 Daehak-ro, Yuseong-gu, Daejeon 34134, Korea; 4 Department of Mechanical Engineering, Korea Advanced Institute of Science and Technology, 291 Daehak-ro, Yuseong-gu, Daejeon 34141, Korea; 5 School of Electrical Engineering, Korea Advanced Institute of Science and Technology, 291 Daehak-ro, Yuseong-gu, Daejeon 34141, Korea

**Keywords:** depth completion, local similarity, knowledge distillation, model compression, sensor fusion, multimodal learning

## Abstract

Depth perception capability is one of the essential requirements for various autonomous driving platforms. However, accurate depth estimation in a real-world setting is still a challenging problem due to high computational costs. In this paper, we propose a lightweight depth completion network for depth perception in real-world environments. To effectively transfer a teacher’s knowledge, useful for the depth completion, we introduce local similarity-preserving knowledge distillation (LSPKD), which allows similarities between local neighbors to be transferred during the distillation. With our LSPKD, a lightweight student network is precisely guided by a heavy teacher network, regardless of the density of the ground-truth data. Experimental results demonstrate that our method is effective to reduce computational costs during both training and inference stages while achieving superior performance over other lightweight networks.

## 1. Introduction

Recent advances in autonomous driving technologies have realized commercial self-driving platforms operating in dynamic real-world environments [1,2]. These real-world systems often benefit from various sensors, such as color cameras, radars, LiDARs, ultrasonic sensors, and thermal cameras, for robust perception in changing environments [3,4,5]. However, the computational cost typically increases with the increasing number of sensors. This problem is critical to commercial platforms because these systems strictly require real-time performance for reliable and robust operation in real-world environments. To ensure real-time performance, existing systems utilize high-cost custom processing units or lightweight perception agents with reduced computational costs but limited performance [6,7].

Among them, robust depth perception is one of the most important tasks for autonomous platforms. A LiDAR is the most popular sensor for accurate depth perception in both indoor and outdoor environments. It provides highly accurate depth measurements from near to far distances; however, it only collects sparse depth values of a scene due to its mechanical and structural limitations. To overcome this limitation, various depth completion algorithms are proposed to combine RGB and LiDAR data because of their complementary characteristics. Ma and Karaman [8] proposed a simple encoder–decoder network for dense depth estimation. A 4-channel image containing RGB and sparse depth is fed into their network for depth estimation. Moreover, spatial propagation algorithms utilizing local and non-local neighbors are proposed to benefit from relevant local information around sparse depth measurement. Cheng et al. [9] presented a convolutional spatial propagation network (CSPN) for depth completion. The CSPN predicts an initial dense depth and it is iteratively refined by a spatial propagation process with local 8-neighbor pixels. Park et al. [10] proposed a non-local spatial propagation network (NLSPN), which utilizes pixel-wise non-local neighbors during the propagation. Unfortunately, the aforementioned algorithms rely on heavy networks that do not ensure real-time performance. To overcome this limitation, lightweight networks for depth completion tasks were proposed. Tao et al. introduced lightweight depth completion with a Sobel edge prediction network [11] and self-attention-based multi-level feature integration and extraction [12]. Although these approaches contribute to decreasing the computational cost by effectively reducing the parameter size and model complexity, they cannot leverage or surpass the better performance of existing networks.

Recently, various knowledge distillation (KD) methods have been proposed to consider the balance between high performance and computational costs. They aim to maintain the robust performance of heavy networks while reducing computational costs and network sizes based on the concept of teacher and student networks. For instance, a heavy teacher network is trained with large-scale datasets, and then a lightweight student network is trained with both large-scale (or small-scale) datasets and precise guidance from the teacher network. With the KD, the lightweight student can achieve better performance compared to the student trained without guidance from the teacher. Therefore, various KD methods have been proposed for numerous low- to high-level perception tasks recently. Xu et al. [13] proposed logit, feature, and structure distillations for human pose estimation. Liu et al. [14] adopted KD for video-based egocentric activity recognition. Yoon et al. [15] proposed spatial- and channel-wise similarity-preserving KD for image matting problems. Yang et al. [16] proposed a cross-image relation KD for semantic segmentation problems. However, typical KD methods require large computational resources during the distillation. Therefore, distillation is often conducted with high-level features requiring small computing resources, although distillation on low-level features is proven to be more effective [15].

In order to benefit from lightweight network architectures with low- to high-level distillation, in this paper, we propose local similarity-preserving knowledge distillation (LSPKD) for depth completion. Previous KD methods [15,17] have demonstrated that the intra-similarity of features can accurately guide student networks during the distillation. However, they utilize global similarity, consuming large computing resources, while local information is more beneficial in various depth completion methods [9,10]. Based on this observation, we propose to focus on local similarity preservation for reduced computational costs during both distillation and inference. With our LSPKD, a lightweight student network achieves superior performance compared to those trained with conventional distillation methods or without distillations.

## 2. Method

In this section, we first describe the baseline teacher and student architectures for the depth completion. Afterwards, the proposed local similarity-preserving KD is presented.

### 2.1. Problem Formulation

A dense depth map *D* can be predicted from network *g* with a sparse depth map $D^{\prime }$ with parameter θ [18,19].

(1) $$D=g(D^{\prime };\theta )$$

Due to the sparse nature of typical LiDAR point clouds, it is important to combine local information from the paired color image around these points for accurate dense depth estimation. If a corresponding RGB image *I* whose pixels are aligned with $D^{\prime }$ is utilized as a guide for input sparse depth, (1) can be formulated by

(2) $$D=g(D^{\prime },I;\theta ).$$

The parameter θ can be optimized to train the network by minimizing loss function L with given ground-truth depth $D_{gt}$.

(3) $$\theta^{*}=\underset{\theta }{\mathrm{argmin}}L\left(g\left(D^{\prime },I;\theta \right),D_{gt}\right)$$

The learning problem is to determine $\theta^{*}$ with effectively designed loss function L. Predicted depth maps are evaluated based on metrics such as RMSE, MAE, iRMSE, and iMAE [3] to estimate performance. Moreover, the size of parameter θ mainly affects the computational cost.

### 2.2. Network Architecture

**Figure 1:**
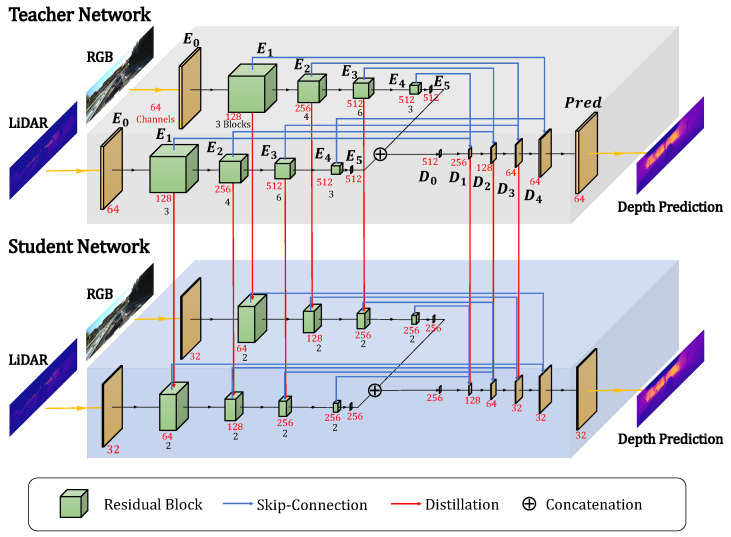
An overall pipeline of the proposed algorithm. The ResNet34-based teacher network consists of two separate encoders for RGB and LiDAR and a decoder for depth prediction. Output feature dimensionalities of each layer are shown together. Encoder features from RGB and LiDAR are concatenated and fed into the decoder. Skip connections deliver encoder features to decoder layers by concatenation. The ResNet18-based student network is distilled with the knowledge from the teacher network.

Various methods have adopted the convolutional neural network [20] and encoder–decoder network architecture with skip connections [8,9,10,21,22] to solve depth completion problems. In this work, we utilize a ResNet34-based network [23] with skip connections as our teacher network for fair comparison. The teacher network comprises two encoders for RGB and LiDAR and one decoder to fuse multi-modal high-level features. Each encoder has an input convolutional layer, 16 successive basic residual blocks [23], and the last convolutional layer. High-level features extracted from encoders are concatenated to be fed into the decoder that consists of 6 deconvolutional layers. The output feature of each decoder layer is concatenated with corresponding RGB and LiDAR encoder features by skip connections, and then fed into the next decoder layer. Figure 1 shows the overall architecture of our baseline teacher network.

For the student network, we halve the number of basic blocks of the encoders (i.e., ResNet18 [23]) and reduce the number of channels in all the layers of the encoders and decoder. Exact parameter comparisons will be provided for each experimental result separately.

### 2.3. Local Similarity-Preserving Knowledge Distillation

Hinton et al. have shown that it it possible to transfer knowledge from a large model into a smaller, distilled model and demonstrated that the knowledge distillation (KD) method is applicable for not only image classification but also commercial acoustic model systems [24]. Similarity-preserving KD algorithms [15,17] have demonstrated their effectiveness in various applications, such as classification and image matting. These tasks are suited to exploiting inter-image similarity [17] or global intra-image similarity [15].

However, many depth completion works [9,10] make use of local and non-local information around depth measurements rather than the global information across the entire image due to the geometric nature of natural scenes. In other words, a local area in a scene typically has continuous depth values, except for object boundaries. Moreover, measuring global similarity across the entire image consumes a huge amount of GPU memory during the distillation process [15]. Therefore, conventional methods usually search for a subset of layers of the network to distill due to the limited computational resources.

With this observation, we propose a local similarity-preserving KD to effectively utilize the similarity information of low-level features without huge memory requirements during the distillation process. We first calculate the local similarity of a reference feature to its neighbors as follows:

(4) $$S\left(x,y,j\right)=f{\left(x,y\right)}^{⊤}\cdot f\left(x+p_{j},y+q_{j}\right),$$

where *f* denotes the $\ell_{2}$ normalized feature, *x* and *y* are the reference pixel coordinates, *j* is the index of the neighbors, and $p_{j}$ and $q_{j}$ are pixel offsets of the *j*-th neighbor from the reference, respectively. We adopt the conventional 8-neighbor configuration N for the distillation as follows:

(5) N={(p,q)|p,q∈{−1,0,1},(p,q)≠(0,0)}.

Note that given a feature map $F\in R^{H\times W\times C}$, the local similarity *S* is calculated for each pixel and then we construct $S\in R^{H\times W\times N}$, regardless of the channel dimensionality *C*, where *H*, *W*, and *N* are the height, width, and the number of local neighbors, respectively. Based on the local similarity *S* calculated from paired teacher and student layers, the proposed LSPKD loss is defined as follows:

(6) $$L_{LS}\left(F_{t},F_{s},S_{t},S_{s}\right)={∥F_{t}-\phi \left(F_{s}\right)∥}_{2}+\alpha {∥S_{t}-S_{s}∥}_{2},$$

where α is a weight parameter and *t* and *s* indicate that *F* and *S* come from the teacher and student networks, respectively. $\phi \left(\cdot \right)$ is a dimensionality matching function between teacher and student features in case their channel numbers are different. We adopt a 1 × 1 convolutional layer as $\phi \left(\cdot \right)$ for efficiency. The proposed $L_{LS}$ consists of two components. The first term enforces pixel-level feature similarity (with auxiliary dimensionality matching) to directly distill features extracted from the deep network. This direct distillation is simple but effective in transferring valuable knowledge from the teacher to the student [25]. The second term further improves the student by enforcing it to preserve the local similarity of the teacher network. Note that the local similarity is closely related to the affinity, which is proven to be highly effective in densifying predictions for various applications [10,26,27].

### 2.4. Training Lightweight Depth Completion Network

To train the lightweight student network, we utilize both the dense depth prediction from the teacher and the ground truth (GT). Let $D_{gt}$, $D_{t}$, and $D_{s}$ be the GT and predictions from the teacher and student networks, respectively. The student prediction $D_{s}$ can be supervised with $D_{t}$ and $D_{gt}$ as follows:

(7) $$L_{gt}\left(D_{gt},D_{s}\right)={∥D_{gt}-D_{s}∥}_{1},$$

(8) $$L_{pred}\left(D_{t},D_{s}\right)={∥D_{t}-D_{s}∥}_{1},$$

where $\ell_{1}$ loss is adopted for better depth boundary predictions.

The final loss function is defined as follows:

(9) $$L_{distill}=L_{gt}+w_{1}L_{pred}+w_{2}L_{LS},$$

where $w_{1}$ and $w_{2}$ are user parameters.

## 3. Experiments

In this section, we describe the implementation details of the proposed LSPKD. Then, we present quantitative and qualitative evaluations on two public depth completion benchmark datasets [3,28], as well as in-depth analyses. Moreover, we present the impact of layer selection for knowledge distillation by providing a comparison of performance among the results of various layer combinations. Robustness to the sparsity of the supervision signal is presented to verify the effectiveness of our algorithm.

### 3.1. Implementation Details

Our algorithm is implemented using the PyTorch framework [29] on a machine equipped with two NVIDIA V100 GPUs. For the training, the ADAM optimizer is used with the initial learning rate 0.001, $\beta_{1}=0.9$, and $\beta_{2}=0.999$. For all the experiments, we set $\alpha =w_{1}=1$. We follow conventional depth completion works [8,9,10] and adopt RMSE (mm), MAE (mm), iRMSE (1/km), iMAE (1/km), REL, and $\delta_{t}$ for our evaluation metrics. More detailed configurations will be described for each dataset in the following sections.

For the distillation, we adopt probabilistic knowledge transfer (PROB) [30] and attention transfer (ATT) [31] for comparisons. These methods are adopted because they introduce small additional computational burdens during the distillation. Implementation details for layer combinations for the distillation will be explained in Section 3.4 in detail.

### 3.2. KITTI Depth Completion

The KITTI Depth Completion (KITTI DC) dataset [32] provides approximately 86K RGB and LiDAR depth images for the training and 7K images for the validation, respectively. The teacher and student networks are trained for 20 epochs with 8 and 16 batch sizes, respectively. For the student network, we halved the number of channels in all the layers and set $w_{2}=1$. As a result, the student network has approxiately 16.53% parameters compared to those of the teacher network.

**Figure 2:**
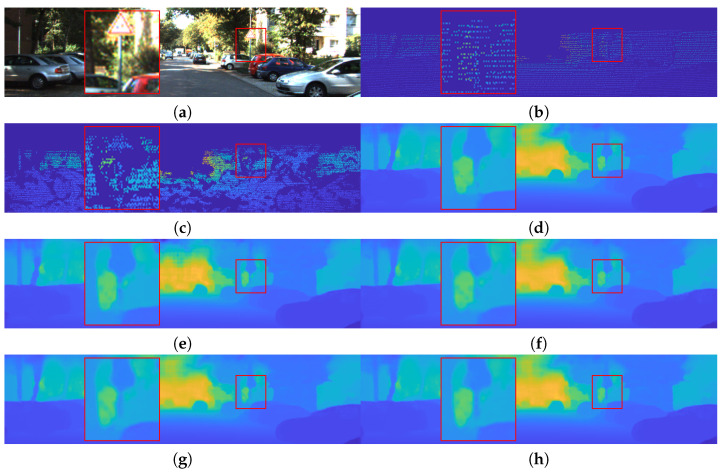
Depth prediction results on the KITTI DC validation dataset [32]. (**a**) RGB. (**b**) Sparse Depth. (**c**) GT. (**d**) Teacher. (**e**) Student. (**f**) PROB [30]. (**g**) ATT [31]. (**h**) Ours.

Table 1 shows quantitative evaluation results on the KITTI DC validation set, as well as the number of parameters and FLOPs. We adopted Self S2D [33] for comparison because it has the same baseline architecture. Note that our teacher network has more parameters because of the individual encoders for the RGB and LiDAR branches. However, due to the progressive downsampling of features, our network requires fewer computational operations. As reported in Table 1, our teacher network shows better performance compared to Self S2D. The small student network trained from scratch shows poor performance, as expected. However, with various distillations, including PROB [30] and ATT [31], the small network achieves a substantial performance improvement. Furthermore, the proposed LSPKD outperforms both PROB and ATT. In addition, LSPKD can be seamlessly combined with PROB and ATT to further improve the performance. We argue that the reason for the superiority of the LSPKD is that the local information is highly important in depth completion tasks. Figure 2 shows qualitative comparisons on the KITTI DC dataset. Compared to the other methods, our method successfully preserves fine depth structures for dense prediction.

### 3.3. NYU Depth V2

The NYU Depth V2 (NYUv2) dataset [28] consists of approximately 50K RGB and depth images for the training and 1.5K images for the evaluation, respectively. The teacher and student networks are trained for 15 epochs with a batch size of 32, similarly to the KITTI DC dataset configuration. For the student network, the number of channels in all the layers is reduced to 1/8 (i.e., 1.30% parameters) and $w_{2}$ is set to 0.1.

Table 2 provides quantitative evaluations on the NYUv2 validation set. Due to the significantly reduced number of parameters, PROB [30] failed to improve the student network (i.e., worse performance than the student trained from scratch). Contrarily, the proposed LSPKD successfully distilled the student network and outperformed the naive student network. Different from the KITTI DC case, combining conventional algorithms does not always lead to improved performance in the NYUv2. Therefore, we conclude that our LSPKD is sufficient for highly lightweight network distillation.

### 3.4. Ablation Studies

In this subsection, we provide analyses of the impact of layer selection for distillation and robustness to the sparsity of the supervision signal to verify the effectiveness of our algorithm.

#### 3.4.1. Layer Selection for Distillation

The effectiveness of the distillation on each layer of a deep network can vary drastically depending on the network architecture or target tasks. Table 3 shows performance comparison results with various combinations of layers for the distillation. Overall, the distillation performance is poor when using only the layers in the encoder. Moreover, the performance is degraded when using only the high-level feature layers of the encoder and decoder (i.e., $\left\{E_{2},E_{2},E_{4}\right\}$ and $\left\{D_{0},D_{1},D_{2}\right\}$ in Figure 1). In contrast, mid-level layers (i.e., $\left\{E_{1},E_{2},E_{3}\right\}$ and $\left\{D_{1},D_{2},D_{3}\right\}$ in Figure 1) have shown a substantial performance improvement when used for the distillation. We presume that the similarities of very low-level or very high-level layers provide limited local or overly wide-range information that is not suitable for depth completion. Thus, we have adopted $\left\{E_{1},E_{2},E_{3}\right\}$ and $\left\{D_{1},D_{2},D_{3}\right\}$ for the distillation for all experiments.

#### 3.4.2. Sparsity of Supervision

The KITTI DC dataset provides semi-dense ground-truth depth data for the training by accumulating a number of successive frames to the reference frame with outlier filtering. The density (i.e., precision) of the GT can vary depending on how many frames are accumulated. Therefore, this level of GT density is often not available in various real-world scenarios. In the extreme case, there may be only one frame to produce the GT depth data, in which case only very sparse depth data (e.g., exactly the same as the input LiDARs) are available. Therefore, we validate the effectiveness of our method with highly sparse supervision signals (i.e., self-supervision with input LiDARs).

We trained the student network with very sparse depth data instead of GT ones. Note that the teacher network is trained by GT and its parameters are fixed during the distillation. Each distillation method achieved the following RMSE: {Naive student: 16140.7, PROB [30]: 1185.4, ATT [31]: 1197.3, Ours: 1179.0}. Note that the density of sparse supervision decreases to 9.1% of the semi-dense GT; therefore, the naive student failed to converge and the overall performance is decreased for all methods. However, our method still achieves the best performance compared to the others. This result empirically demonstrates that our LSPKD is robust to the density of supervision signals thanks to the local similarities.

#### 3.4.3. Comparison to Global Similarity-Preserving KD

We compare the proposed LSPKD with a global similarity-preserving KD method (i.e., SPKD [15]) to validate the efficiency and effectiveness of our method. Because the SPKD requires a huge amount of memory to distill low- and mid-level features, we have distilled $\left\{E_{2},E_{3},E_{4}\right\}$ and $\left\{D_{0},D_{1},D_{2}\right\}$ for comparison with the batch size 12, and we obtained the following RMSE and GPU memory consumption for the training per image: {SPKD: 901.6/7.2 GB, LSPKD: 903.6/1.70 GB, LSPKD (Mid-level): 893.0/1.71 GB}. Note that our method shows comparable performance to the SPKD, and outperforms it with the mid-level feature distillation. Low- or mid-level distillations are possible only for our LSPKD because the GPU memory requirement is significantly smaller compared to that of the original SPKD. Therefore, we conclude that our method is suitable for distilling low- or mid-level features without enormous GPU memory requirements for both training and inference for efficiency and performance improvement.

## 4. Conclusions

In this paper, we have proposed a lightweight depth completion network with local similarity-preserving knowledge distillation. A lightweight depth completion network is effectively trained by the proposed distillation algorithm, with low computational costs for both training and inference stages. The trained network maintains performance comparable to that of previous depth completion networks and superior to the performance of a student network without distillation. Additionally, the experimental result shows that our LSPKD outperforms previous distillation algorithms in both indoor and outdoor datasets. Moreover, the proposed method is verified to be robust to the density level of the supervision signals. For future works, various similarity metrics can be considered for the local similarity estimation.

## Figures and Tables

**Table 1:** Quantitative evaluation results on the KITTI DC validation dataset [32] (T: Teacher, S: Student, D: Distilled).

Network	# Params (M)/GFLOPs (912 × 220)	Distillation	Metrics			
			RMSE	MAE	iRMSE	iMAE
Self S2D [33]	26.11/637.89	-	878.6	260.9	3.3	1.3
ResNet34 (T)	51.77/349.36	-	865.2	222.1	2.4	1.0
ResNet18 (S)	8.56/59.22	-	921.5	233.3	2.7	1.0
ResNet18 (D)	8.56/59.22	PROB [30]	902.6	243.3	8.5	1.1
		ATT [31]	907.6	245.0	2.7	1.1
		Ours	893.0	234.9	2.8	1.0
		Ours + PROB	893.7	238.6	2.6	1.0
		Ours + ATT	893.3	243.5	2.6	1.0
		Ours + PROB + ATT	891.8	238.6	2.7	1.0

**Table 2:** Quantitative evaluation results on the NYUv2 validation dataset [28] (T: Teacher, S: Student, D: Distilled).

Network	# Params (M)/GFLOPs (304 × 228)	Distillation	Metrics				
			RMSE	REL	$\delta_{1.25}$	$\delta_{1.25^{2}}$	$\delta_{1.25^{3}}$
S2D + SPN [8,27]	31.88/24.53	-	172.0	0.0310	0.9710	0.9940	0.9980
DeepLiDAR [34]	143.98/502.12	-	115.0	0.0220	0.9930	0.9990	1.0000
ResNet34 (T)	51.77/112.32	-	114.4	0.0184	0.9932	0.9989	0.9998
ResNet18 (S)	0.66/1.46	-	152.1	0.0282	0.9875	0.9978	0.9995
ResNet18 (D)	0.66/1.46	PROB [30]	154.8	0.0328	0.9891	0.9982	0.9996
		ATT [31]	149.9	0.0302	0.9891	0.9982	0.9996
		Ours	138.8	0.0248	0.9899	0.9984	0.9997
		Ours + PROB	138.9	0.0249	0.9900	0.9984	0.9997
		Ours + ATT	138.7	0.0248	0.9899	0.9984	0.9997
		Ours + PROB + ATT	143.6	0.0268	0.9899	0.9984	0.9997

**Table 3:** Performance comparison with various combinations of layers for the distillation on the KITTI DC validation dataset [32].

Encoder					Decoder					Metrics			
**E0**	**E1**	**E2**	**E3**	**E4**	**D0**	**D1**	**D2**	**D3**	**D4**	**RMSE**	**MAE**	**iRMSE**	**iMAE**
-	-	✓	✓	✓	-	-	-	-	-	899.3	241.8	2.7	1.1
-	-	-	-	-	✓	✓	✓	-	-	897.1	239.9	2.9	1.0
-	✓	✓	✓	-	-	-	-	-	-	894.4	235.5	2.5	1.0
-	-	-	-	-	-	✓	✓	✓	-	896.4	237.9	2.6	1.0
✓	✓	✓	-	-	-	-	-	-	-	901.7	239.4	2.8	1.1
-	-	-	-	-	-	-	✓	✓	✓	899.8	242.0	2.6	1.0
✓	✓	✓	✓	-	-	-	-	-	-	898.1	237.2	2.6	1.0
-	-	-	-	-	-	✓	✓	✓	✓	894.1	238.3	2.6	1.0
-	-	✓	✓	✓	✓	✓	✓	-	-	902.8	236.7	2.7	1.0
-	✓	✓	✓	-	-	✓	✓	✓	-	893.0	234.9	2.8	1.0
✓	✓	✓	-	-	-	-	✓	✓	✓	898.7	236.8	2.6	1.0
✓	✓	✓	✓	-	-	✓	✓	✓	✓	894.9	235.7	2.6	1.0

## References

[1] Ettinger S., Cheng S., Caine B., Liu C., Zhao H., Pradhan S., Chai Y., Sapp B., Qi C.R., Zhou Y. Large Scale Interactive Motion Forecasting for Autonomous Driving: The Waymo Open Motion Dataset. Proceedings of the IEEE/CVF International Conference on Computer Vision (ICCV).

[2] Wilson B., Qi W., Agarwal T., Lambert J., Singh J., Khandelwal S., Pan B., Kumar R., Hartnett A., Pontes J.K. Argoverse 2: Next Generation Datasets for Self-Driving Perception and Forecasting. Proceedings of the Thirty-Fifth Conference on Neural Information Processing Systems Datasets and Benchmarks Track (Round 2).

[3] Geiger A., Lenz P., Urtasun R. Are we ready for autonomous driving? The kitti vision benchmark suite. Proceedings of the Computer Vision and Pattern Recognition (CVPR).

[4] Fong W.K., Mohan R., Hurtado J.V., Zhou L., Caesar H., Beijbom O., Valada A. (2022). Panoptic nuscenes: A large-scale benchmark for lidar panoptic segmentation and tracking. IEEE Robot. Autom. Lett..

[5] Malawade A.V., Mortlock T., Al Faruque M.A. HydraFusion: Context-Aware Selective Sensor Fusion for Robust and Efficient Autonomous Vehicle Perception. Proceedings of the 2022 ACM/IEEE 13th International Conference on Cyber-Physical Systems (ICCPS).

[6] Zheng W., Tang W., Jiang L., Fu C.W. SE-SSD: Self-Ensembling Single-Stage Object Detector from Point Cloud. Proceedings of the IEEE/CVF Conference on Computer Vision and Pattern Recognition (CVPR).

[7] Chen Q., Wang Y., Yang T., Zhang X., Cheng J., Sun J. You Only Look One-Level Feature. Proceedings of the IEEE/CVF Conference on Computer Vision and Pattern Recognition (CVPR).

[8] Ma F., Karaman S. (2018). Sparse-to-dense: Depth prediction from sparse depth samples and a single image. Proceedings of the IEEE International Conference on Robotics and Automation (ICRA).

[9] Cheng X., Wang P., Yang R. Depth estimation via affinity learned with convolutional spatial propagation network. Proceedings of the European Conference on Computer Vision (ECCV).

[10] Park J., Joo K., Hu Z., Liu C.K., Kweon I.S. Non-Local Spatial Propagation Network for Depth Completion. Proceedings of the European Conference on Computer Vision (ECCV).

[11] Tao Z., Shuguo P., Hui Z., Yingchun S. (2021). Dilated U-block for lightweight indoor depth completion with sobel edge. IEEE Signal Process. Lett..

[12] Zhao T., Pan S., Gao W., Sheng C., Sun Y., Wei J. (2022). Attention Unet++ for lightweight depth estimation from sparse depth samples and a single RGB image. Vis. Comput..

[13] Xu X., Zou Q., Lin X., Huang Y., Tian Y. (2020). Integral knowledge distillation for multi-person pose estimation. IEEE Signal Process. Lett..

[14] Liu T., Zhao R., Xiao J., Lam K.M. (2020). Progressive Motion Representation Distillation With Two-Branch Networks for Egocentric Activity Recognition. IEEE Signal Process. Lett..

[15] Yoon D., Park J., Cho D. (2020). Lightweight deep cnn for natural image matting via similarity-preserving knowledge distillation. IEEE Signal Process. Lett..

[16] Yang C., Zhou H., An Z., Jiang X., Xu Y., Zhang Q. Cross-image relational knowledge distillation for semantic segmentation. Proceedings of the IEEE/CVF Conference on Computer Vision and Pattern Recognition.

[17] Tung F., Mori G. Similarity-preserving knowledge distillation. Proceedings of the International Conference on Computer Vision (ICCV).

[18] Qu C., Nguyen T., Taylor C. Depth Completion via Deep Basis Fitting. Proceedings of the IEEE/CVF Winter Conference on Applications of Computer Vision (WACV).

[19] Hu J., Bao C., Ozay M., Fan C., Gao Q., Liu H., Lam T.L. (2022). Deep Depth Completion from Extremely Sparse Data: A Survey. arXiv.

[20] Chen L., Li Q. (2022). An Adaptive Fusion Algorithm for Depth Completion. Sensors.

[21] Lee B.U., Jeon H.G., Im S., Kweon I.S. (2019). Depth completion with deep geometry and context guidance. Proceedings of the IEEE International Conference on Robotics and Automation (ICRA).

[22] Lee B.U., Lee K., Kweon I.S. Depth Completion using Plane-Residual Representation. Proceedings of the Computer Vision and Pattern Recognition (CVPR).

[23] He K., Zhang X., Ren S., Sun J. Deep residual learning for image recognition. Proceedings of the Computer Vision and Pattern Recognition (CVPR).

[24] Hinton G., Vinyals O., Dean J. Distilling the Knowledge in a Neural Network. Proceedings of the Neural Information Processing Systems Workshops (NeurIPSW).

[25] Romero A., Ballas N., Kahou S.E., Chassang A., Gatta C., Bengio Y. Fitnets: Hints for thin deep nets. Proceedings of the International Conference on Learning Representations.

[26] Levin A., Lischinski D., Weiss Y. (2007). A closed-form solution to natural image matting. IEEE Trans. Pattern Anal. Mach. Intell..

[27] Liu S., De Mello S., Gu J., Zhong G., Yang M.H., Kautz J. (2017). Learning affinity via spatial propagation networks. Proc. Adv. Neural Inf. Process. Syst..

[28] Silberman N., Hoiem D., Kohli P., Fergus R. Indoor Segmentation and Support Inference from RGBD Images. Proceedings of the European Conference on Computer Vision (ECCV).

[29] Paszke A., Gross S., Massa F., Lerer A., Bradbury J., Chanan G., Killeen T., Lin Z., Gimelshein N., Antiga L. (2019). Pytorch: An imperative style, high-performance deep learning library. Proc. Adv. Neural Inf. Process. Syst..

[30] Passalis N., Tefas A. Learning deep representations with probabilistic knowledge transfer. Proceedings of the European Conference on Computer Vision (ECCV).

[31] Zagoruyko S., Komodakis N. Paying more attention to attention: Improving the performance of convolutional neural networks via attention transfer. Proceedings of the International Conference on Learning Representations.

[32] Uhrig J., Schneider N., Schneider L., Franke U., Brox T., Geiger A. (2017). Sparsity invariant cnns. Proceedings of the International Conference on 3D Vision (3DV).

[33] Ma F., Cavalheiro G.V., Karaman S. (2019). Self-supervised sparse-to-dense: Self-supervised depth completion from lidar and monocular camera. Proceedings of the IEEE International Conference on Robotics and Automation (ICRA).

[34] Qiu J., Cui Z., Zhang Y., Zhang X., Liu S., Zeng B., Pollefeys M. Deeplidar: Deep surface normal guided depth prediction for outdoor scene from sparse lidar data and single color image. Proceedings of the Computer Vision and Pattern Recognition (CVPR).

